# Parental Depressive Symptoms, Parent Attributional Style, and Child Coping as Predictors of Depressive Symptoms in Children of Parents with Anxiety or Mood Disorders

**DOI:** 10.1007/s10578-021-01248-w

**Published:** 2021-09-21

**Authors:** Mun Wong, Thomas G. Power

**Affiliations:** 1 Department of Early Childhood Education, The Education University of Hong Kong, 10 Lo Ping road, Tai Po, New territories, Hong Kong; 2 Department of Human Development, Washington State University, Pullman, WA USA

**Keywords:** Child depression, Parental depression, Parental anxiety, Parental attributional style, Coping

## Abstract

Few studies have examined the effects of parental depressive symptoms on children in China. The present study examined the relationships between parental depression, parental attributional style, children’s coping strategies and 5–12-year-old children’s depressive symptoms in a sample of Chinese children whose parents had been diagnosed with an anxiety or a mood disorder. The present study confirmed that children of parents with anxiety or mood disorders would show high levels of depressive symptoms. Parents with an optimistic or neutral attributional style rated their children as showing fewer depressive symptoms than parents with a pessimistic style. This study showed a significant positive relationship between children’s disengagement coping and children’s reports of depressive symptoms. The findings highlight the need for early identification of, and support and intervention programs for, parents suffering from depression and children of depressed parents as a means of protecting the psychological well-being of both parents and children.

## Introduction

Research shows that parent’s depression and anxiety can negatively affect school age children’s mental and physical health [1–3]. In a recent review of the literature on parental psychopathology and child development, Suchman and colleagues [4] cited numerous studies showing that during the school-age years, parental depression and anxiety are associated with negative patterns of parenting (e.g., poor communication, low responsiveness, and critical/harsh/controlling parenting behavior) and a wide range of child adjustment problems including poor academic performance, poor peer relationships, and a range of internalizing and externalizing symptoms. Goodman and colleagues [5], in a meta-analysis examining maternal depression and children’s psychopathology, reported an effect size of *r* = 0.23 across the 121 studies that examined children’s internalizing symptoms. The highest effect sizes were found when: (1) depression was diagnosed with a clinical interview versus symptom ratings; (2) mothers were recruited from clinic versus community settings; (3) child depressive symptoms were rated by the mother versus the child; and (4) mothers had low- versus middle-income backgrounds.

The vast majority of studies on this issue, however, have employed samples from the U.S. or Europe. Several factors make it unclear whether these findings would be replicated in a Chinese context. For example, in comparison to Western settings, Chinese adults often suppress distressing emotions, because people may worry that an open discussion of personal distress outside the family may result in social stigmatization that could bring disgrace to the family [6]. As a result, depressed adults in China are more likely than Western adults to report more somaticizing symptoms such as headaches and poor appetite than depressed mood [6–8] and Chinese adults are less likely to seek professional help for their condition [9]. Finally, the relationships between certain childrearing practices and child outcomes (particularly coercive authority assertion and critical comparison/shaming—practices often associated with maternal depression—[4]) often differ for North American and Chinese families depending on the child’s approval of these practices [10]. In a study of undergraduate students, Chinese students, compared to Australians, reported higher levels of perceived stigma, emphasized somatic symptoms, minimized depressive symptoms, and suppressed emotional expression [6]. Given these differences in the symptom profile of depressive disorders, the nature of parent–child relationships, the nature of emotional expression, and larger cultural values and practices, it is not clear that the frequently identified relationships between parent and child depressive symptoms found in Western settings would be replicated in a Chinese context. Wang [11], in the only study of school age Chinese children we could locate on this topic, found that the correlation between parents’ reports of their own depressive symptoms on the Center for Epidemiologic Studies Depression Scale (CES-D) [12] and parents’ ratings of childhood depressive symptoms on a subset of items from the Child Behavior Checklist [13] was positive (*r* = 0.26). This value is consistent with the value of *r* = 0.25 that Goodman and colleagues [5] reported in their meta-analysis for those 68 studies using parent reports of childhood internalizing symptoms.

Although Wang [11] presents some valuable initial data on the relationship between parents’ and children’s depressive symptoms in China, the use of only parent ratings to assess children’s depressive symptoms can be problematic because the parents’ own depression may lead them to focus on negative aspects of their child’s behavior and thereby overestimate the level of children’s depressive symptoms [14]. On the other hand, children might be hesitant to report depressive symptoms on self-report questionnaires because they want to present themselves in a favorable light [15]. Because the average correlation between parents’ ratings of their children’s depressive (or internalizing) symptoms and children’s reports of their own symptoms is *r* = 0.26 [14, 16], researchers have argued that the best approach for assessing children’s depressive symptoms is to use multiple raters, providing overlapping, yet somewhat different, perspectives on children’s adjustment yielding a better assessment than relying on parent or child ratings alone [17, 18]. In the current study, we examined the relationship between parents’ and children’s depressive symptoms using both parent and child ratings.

The relationship between parents’ and children’s depressive symptoms is typically stronger for parents recruited from clinic versus community settings [5]. This is probably due to the more limited range of depressive symptoms found in community samples. Depressive symptoms are common in children of parents who have been diagnosed with unipolar or bipolar depression. Downey and Coyne [19] summarized the results of nine studies comparing the prevalence of clinically diagnosed affective disorders in children of clinically depressed mothers versus healthy mothers. Between 21 and 74% of the children of depressed mothers in these studies had been diagnosed with an affective disorder (*M* = 45%). More recent studies show similar rates: Thompson et al.[20] (2010—24%) and Gershon et al.[21] (2011—32%). Given that previous studies have not examined the rates of depressive symptoms in children of clinically depressed Chinese parents, a second purpose of this study was to provide such data.

Despite being at genetic risk for developing depressive symptoms, not all children of clinically depressed mothers develop depressive symptoms [4]. Factors that can increase or decrease the likelihood of developing symptoms include the timing, nature, and course of the maternal depression, environmental stresses, marital discord, the child’s relationships with other family members (e.g., fathers and siblings), and child characteristics (e.g., temperament or social-cognitive skills) [2, 4, 19]. Because numerous studies have shown that a pessimistic attributional style in adults is associated with later depressive symptoms [22], we explored, in this study, the possibility that parents who were able to maintain an optimistic or neutral attributional style, despite their anxiety or mood disorder, would have children with the lowest levels of depressive symptoms. Because attributional style can operate as a moderating variable [23], we empirically tested the possibility that the association between parental and children’s depressive symptoms would be strongest for parents with a pessimistic attributional style.

Finally, we examined the role of child coping as protective factor against the stresses of being raised by a clinically depressed parent. Research shows that the ability to effectively cope with stress significantly lowers the risk of psychological distress and symptoms [24, 25]. The current study examined children’s coping based on Lazarus and Folkman’s [26] model using a questionnaire developed based on this theory–Kid-cope [27]. In a previous study of children during their first year of primary school in Hong Kong [28], a principal components analysis of this questionnaire yielded two types of coping consistent with the Lazarus and Folkman [26] and the Connor-Smith et al. [29] coping models: (a) engagement coping including both primary control strategies to change the stressful situation (e.g., problem solving or seeking social support) and secondary control strategies aimed at changing the self to accommodate to stressful conditions (e.g., positive thinking) and (b) disengagement coping (avoidance, denial, wishful thinking, and blaming self or others). In a meta-analysis of studies from a wide range of populations, Compas and colleagues [24] found that engagement coping was usually negatively associated with children’s internalizing symptoms, whereas disengagement coping usually showed a positive (and somewhat larger) association. Distraction (an aspect of secondary control engagement coping in the Connor et al. [29] model) emerged as a separate component in the Wong and Power [28] study. Distraction typically does not show a significant relationship with internalizing symptoms [24]. When examining longitudinally the correlates of children’s coping strategies, Wong and Power [28] found that children’s coping only predicted childhood depressive symptoms for females—engagement coping negatively predicted subsequent depressive symptoms, whereas distraction was a positive predictor. These results are consistent with studies showing sex differences in the relationship between depressive symptoms and coping in older children [30–33] where only females showed significant relationships between engagement and/or disengagement coping strategies and internalizing symptoms.

The current study examined the associations between parental depression, parental attributional style, children’s coping, and depressive symptoms in a Chinese sample of children of parents with anxiety or mood disorders. The data were collected at pretest for the Little Angel Project [34], an intervention using creative arts and drama activities to target children’s coping strategies and depressive symptoms. The aims of the study were to explore:

1. The association of parents’ depressive symptoms and children’ depressive symptoms;
2. The association of parents’ attributional style and children’s depressive symptoms; and
3. The association of children’s coping strategies and children’s depressive symptoms.

Based on the previous findings we hypothesized that:

1. Children of parents with an anxiety or mood disorder would show high levels of depressive symptoms.
2. Parents’ depressive symptoms would be positively associated with children’s depressive symptoms.
3. Parents’ optimistic attributional style would be negatively associated with children’s and parents’ depressive symptoms.
4. The relationship between parents’ and children’s depressive symptoms would be strongest for parents with a pessimistic attributional style.
5. Children’s coping strategies would be significantly associated with children’s depressive symptoms after statistically controlling for parents’ attributional style and depressive symptoms: children’s engagement strategies would be negatively associated with children’s depressive symptoms, whereas disengagement strategies would be positively related. We also predicted that these relationships would only be significant for females.

## Method

### Participants

The parents with mental illness in this study were clients of Caritas Little Angel Project, a pilot service of the Integrated Community Centre for Mental Wellness (ICCMW) in North District in Hong Kong in 2016–19. Ninety-seven mothers and 13 fathers with anxiety or mood disorders and their 5–12-year-old children (M = 8. 4 years, SD = 1.6 years) participated. The ICCMW provides accessible, one-stop, district-based community support and social rehabilitation for persons with mental illnesses and their families. Clients are either self-referred or referred from the psychiatric services of the Hospital Authority (HA). Before clients are admitted to the ICCMW, trained mental health workers conduct clinical interviews and assess presenting problems, mental health symptoms, psychiatric history, and impairments based on the diagnostic criteria of the DSM-5. For example, to assess that a person suffered from depression, the individual must have experienced 5 or more symptoms during the same 2-week period and at least one of the symptoms would be either (1) depressed mood or (2) loss of interest or pleasure.

As can be seen in Table 1, demographic data were available on only 63 parents. For these parents, most were under 46 years old and had completed secondary school. Most mothers were housewives. Parents’ family monthly income ranged mostly from 10,000 to 30,000 HKD (median monthly household income in Hong Kong is 24,400 HKD– [35]). Most children lived with both parents. The diagnoses of parents referred from the Hospital Authority were 51 cases of depression (2 bipolar disorder, 3 postpartum depression), and 15 cases of anxiety disorder. Parents without a diagnosis from Hospital Authority were assessed by a trained mental health worker based on diagnostic criteria of DSM-5 and an additional 44 suspected mood disorder cases (i.e., unipolar or bipolar depression) were included in the study. Comparison of the three diagnostic groups showed that parents in the depression group reported higher levels of depressive symptoms on the CESD-R, *M* = 27.10, *SD* = 14.91, than parents in either the mood disorder, *M* = 17.09, *SD* = 12.36, or anxiety disorder groups, *M* = 15.27, S*D* = 9.42, *F*(2, 107) = 8.58, *p* < 0.001, eta^2^ = 0.14. Follow-up Tukey tests showed that depressive symptoms were significantly higher (*p* < 0.05) for the depression group than for the other 2 groups, who did not significantly differ from one another.

**Table 1:** Demographic information for the participating parents

Characteristic	n	%	% (valid)	M (SD)	Range
Parent sex					
Male	13	11.8	11.8	188 (.324)	
Female	97	88.2	88.2		
Parent age					
25–30	5	4.5	7.9	3.38 (1.361)	
31–35	11	10.0	17.5		
36–40	20	18.2	31.7		
41–45	15	13.6	23.8		
46–50	6	5.5	9.5		
51 or above	6	5.5	9.5		
Missing data	47	42.7			
Total	110	100.0			
Parent occupation					
Full time	20	18.2	31.7	2.32 (1.105)	
Part time	10	9.1	15.9		
Housewife	29	26.4	46.0		
Retired	2	1.8	3.2		
Unemployed	1	.9	1.6		
Others	1	.9	1.6		
Missing data	47	42.7			
Total	110	100.0			
Parent property type					
Public housing estate	29	26.4	46.0	1.87 (0.959)	
Rental property	17	15.5	27.0		
Self-owned property	13	11.8	20.6		
Others	4	3.6	6.3		
Missing data	47	42.7			
Total	110	100.0			
Parent Average family monthly income					
$10,000 or below	13	11.8	20.6	2.76 (1.445)	
$10,001–$20,000	18	16.4	28.6		
$20,001–$30,000	16	14.5	25.4		
$30,001–$40,000	7	6.4	11.1		
$40,001–$50,000	5	4.5	7.9		
$50,001 or above	4	3.6	6.3		
Missing data	47	42.7			
Total	110	100.0			
Parent education level					
Primary school	5	4.5	7.9	3.41 (0.835)	
Secondary school	36	32.7	57.1		
Post secondary/University	13	11.8	20.6		
Post secondary/University or above	9	8.2	14.3		
Missing data	47	42.7			
Total	110	100.0			
Parent family type					
Parental family	34	30.9	54.0	1.89 (1.206)	
Single-parent family	16	14.5	25.4		
Joint family	12	10.9	19.0		
Others	1	.9	1.6		
Missing data	47	42.7			
Total	110	100.0			

#### Measurement Tools

All diagnosed parents completed questionnaires individually and children were individually interviewed by a social worker who read aloud the questions and had children verbally respond.

##### Child Interviews

1. CES-DC (Child Report)

The Center for Epidemiological Studies Depression Scale for Children (CES-DC) was designed to screen children for depressive problems [36]. It is a 20-question self-report instrument with 4-point Likert scales. Each child was asked to choose “not at all” = 0, “A little” = 1, “Some” = 2 and “a lot” = 3 for a sentence during the past week. Total possible scores ranged from 0 to 60; we used the suggested cut-off of greater than 15 to identify children who were likely depressed [36]. Four items were positively worded (e.g., “I had a good time”); the others were negative statements (e.g., “I felt like I was too tired to do things). Research conducted in different countries has validated the CES-DC to measure depression in children and adolescents [37–39]. The alpha coefficient for the Chinese version of the CES-DC was 0.82. Test–retest reliability over a two-week interval was 0.71 (ICC-value). The average equivalence of the semantic rate was 96% and the Content Validity Index was 95%, indicating that each item in the Chinese and English versions remained conceptually and idiomatically the same, showing the CES-DC to be a reliable and valid measure of depressive symptoms in the Chinese context [36]. Wong and Power [40] validated the CES-DC for the assessment of depressive symptoms in Chinese 6-year-old children; the alpha coefficient was 0.87.

2. Kid-Cope Scale

The Kid-cope scale is a 15-item scale (problem-focused, cognitive restructuring, blaming, distraction/avoidance, support-seeking and wishful thinking) measure of children’s coping strategies for use with 5–12-year-olds [27]. Each child responded to 15 coping strategies. For each coping strategy (e.g., “I stayed by myself”), the child was asked to answer “Did you do this coping strategy (yes/no) when you encountered some problems that made you feel upset (e.g., conflict with peers or unhappy events at home). If ‘Yes’, the child evaluated how much it helped (not at all; a little; a lot). The Kid-cope questionnaire has been validated and has an internal reliability coefficient alpha of 0.83 [41]. The Kid-cope scale was also validated in Chinese children [28]. Kid-cope has been widely used to explore children’s coping strategies [42–44].

##### Parents’ Survey

1. CES-DC (parent report of child’s depressive symptoms)

The psychometric properties of the CES-DC (both self- and parent-report) have been empirically tested with good concurrent validity and excellent construct validity and adequate internal consistency (see [36] for a review). The CES-DC (parent report) is designed for asking parents how their children felt during the past week. The content of the CES-DC (parent report) is the same as the CES-DC. It is a 20-question parent-report instrument with 4-point Likert scales. Parents were asked to choose “not at all”, “A little”, “Some” and “a lot” for sentences about how their children have felt during the past week. Four items were positively worded (“Your child was happy”) and the remaining items were negatively worded (“Your child wasn’t able to feel happy, even when family or friends tried to help her feel better”).

2. CESD-R (Adult Version: Self-Evaluation of Depressive Symptoms)

The CESD-R is a revised version of the CES-DC (adult version), developed to reflect modern diagnostic criteria of depressive symptoms. Van Dam and Earleywine [45] have explored psychometric properties of the CESD-R across a large community sample (7389 adults and 245 university students). The result has shown ‘good psychometric properties, including high internal consistency, strong factor loadings, and theoretically consistent convergent and divergent validity with anxiety, schizotypy, and positive and negative affect’ [45]). Results suggest the CESD-R is an accurate and valid measure of depression in the general population. It assesses how people have felt during the past week. It is a 20-item self-report instrument with four-point Likert scales (rarely or none of the time; some or a little of the time; occasional or moderate amount of time; all of the time). The CESD-R (for adults) includes only negative statements (e.g., ‘I felt depressed’, ‘nothing made me happy’). Parents were asked to fill in the CESD-R questionnaires to evaluate their own depressive symptoms. The test–retest reliability of CESD-R support the Chinese version of the CESD-R as a valid and reliable measurement for Chinese samples (intra-class correlation coefficient = 0.73–0.81, all p < 0.001) [46].

3. Adult’s attributional style questionnaire [47, 48]

The ASQ questionnaire includes 16 positive and 16 negative hypothetical situations [48]. For each situation, parents were asked to choose one of two responses (i.e., A or B). For example, “You are asked to head an important project.” (A. I just successfully completed a similar project. B. I am a good supervisor). The ASQ calculates a composite difference score by subtracting positive event scores from negative event ones [48]. Possible scores range from minus below -5 to 16. The higher the scores, the more hopeful would be the person. A person who gets 10 to 16 is extraordinarily hopeful; from 6 to 9, moderately hopeful; from 1 to 5, average hopeful; from minus 5 to 0, moderately hopeless; and below minus 5, severely hopeless [48]. Total ASQ scores of this study ranged from − 5 to 10. To assign parents to attributional styles, parents were assigned to a pessimistic attributional style if their score on the questionnaire was < 0 (n = 37) and to a neutral or optimistic attributional style if the total score was ≥ 0 (n = 71). The ASQ (12-item version) has been validated in a Chinese sample [49].

### Data Analyses

#### Preliminary Analyses

To reduce the number of child coping variables, a principal components analysis with varimax rotation was run on the child coping variables. Component scores were computed by averaging together items loading on the components. Because the response format was dichotomous and the number of items per component small, scale internal consistencies were assessed using mean inter-item correlations rather than coefficient alphas. Based on the recommendations of Clark and Watson [50], mean inter-item correlations between 0.15 and 0.50 were considered acceptable. Frequency distributions of children’s depressive symptoms (both child and parent reports) and parents’ depressive symptoms were run examined and then a 2 × 2 (child sex by reporter) repeated measures analysis of variance (MANOVA approach) examined possible differences in children’s depressive symptoms as a function of child sex and rater. Differences in child depressive symptoms by parent diagnostic group were examined with two-way ANOVAs (diagnostic group by child sex) and follow-up Tukey tests.

#### Tests of Hypotheses

The first hypothesis, that there would be high levels of child depressive symptoms in this sample was examined by calculating the percentage of children who exceeded the clinical cutoffs for depressive symptoms on the child and parent ratings on the CESD. The next two hypotheses, that children’s depressive symptoms would be positively correlated with parents’ depressive symptoms (H2) and negatively correlated with parents’ optimistic attributional style (H3), were examined by computing bivariate correlations. These correlations were also calculated separately for males and females; Fisher’s *z*’ transformation determined if there were significant differences between the correlations for the two sexes.

For hypothesis four, that the relationship between parents’ and children’s depressive symptoms would be strongest for parents with a pessimistic attributional style, we ran separate regressions predicting children’s and parents’ ratings of child depressive symptoms from three predictors: parent depressive symptoms, optimistic/neutral attributional style, and the interaction of parent depressive symptoms and attributional style. Significant interactions were followed up by examining the bivariate correlations separately for parents with an optimistic/neutral or a pessimistic explanatory style. The fifth hypothesis, that children’s coping strategies would be associated with children’s depressive symptoms was tested with separate regressions predicting children’s and parents’ ratings of child depressive symptoms from the following predictors: parent depressive symptoms, parent attributional style, and the measures of child coping derived from the principal components analysis. Additional regressions determined if child coping interacted with parental exploratory style or child sex in predicting child depressive symptoms.

Finally, because 11.8% of the parents in the sample were fathers, we reran the analyses with the data from mothers only to examine the possibility that the relationships between the variables might differ for mothers versus fathers. The analyses showed that the pattern of significant bivariate correlations was identical in both analyses. The regression results were very similar as well. However, 2 of the 16 regression coefficients changed from being statistically significant (*p* < 0.05) in the total sample to nonsignificant (*p* < 0.10 and *p* = 0.14 respectively) in the sample of mothers only (likely due to reduced power). Given the very similar pattern of results across these analyses, we present the results for the total sample to maximize statistical power.

## Results

### Preliminary Analyses

Table 2 presents the results of the principal components analysis of child coping. The scree plot indicated that a 3-component solution was best, accounting for 42.86% of the variance. Two engagement and one disengagement components emerged. One engagement component, “active coping/emotion regulation,” included distraction, problem solving, calming down, wishing things could be different, and seeking social support. The other engagement component, “seeking answers/positive thinking,” had positive loadings for trying to see the good side of things and trying to fix the problem by thinking of an answer and negative loadings for blaming the self or others. “Disengagement coping” included staying by oneself; keeping quiet about the problem; yelling, screaming, or getting mad; wishing the problem had never happened; and doing nothing because the problem could not be fixed. The mean inter-item correlations (0.23, 0.23, 0.25) fell within Clark and Watson’s [50] recommended range of 0.15–0.50.

**Table 2:** Rotated component matrix of the children’s coping responses on children’s coping strategies checklist

Coping Item	Loadings		
	Active coping/emotion regulation	Disengagement coping	Seeking answers/positive thinking
Try to forget	.36	.19	.28
Did something else to forget	*.53*	− .14	.13
Stayed by myself	− .10	*.44*	.12
Kept quiet about the problem	− .14	*.70*	− .09
Tried to see the good side of things	.34	08	*.54*
Blamed myself for causing the problem	.23	.45	− *.51*
Blamed someone else for causing the problem	.02	.23	− *.69*
Tried to fix the problem by thinking of an answer	.23	.16	*.69*
Tried to fix the problem by doing something or talking to someone	*.63*	− .15	.11
Yelled, screamed, or got mad	− .10	*.68*	− .28
Tried to calm myself down	*.47*	− .06	.39
Wished the problem had never happened	.22	*.62*	.11
Wished I could make things different	*.56*	*.*28	.12
Tried to feel better by spending time with others	*.67*	− .11	− .15
Didn’t do anything because the problem couldn’t be fixed	− .35	*.42*	− .32
Percentage of variance accounted for	20.44	14.03	8.39

### Tests of Hypotheses

Presented in Table 3 are the frequency distributions of the various measures of depressive symptoms. Although parents reported higher levels of child depressive symptoms (*M* = 28.94, *SD* = 9.69) than did children (*M* = 21.65, *SD* = 9.68), most children exceeded the clinical cut-off of 15, regardless of reporter: 72.1% for child reports and 91.0% for parent reports. The difference between parent and child reports of child depressive symptoms was significant, *F*(1,108) = 30.77, *p* < 0.001, eta^2^ = 0.22. Neither child nor parent ratings of child depressive symptoms differed by sex or across the three parent diagnostic groups.

**Table 3:** Percentage of children or parents showing various scores on the center for epidemiological studies depression scale

CESD score	Child reports child symptoms	Parent reports child symptoms	Parent reports parent symptoms
0–5	2.7	0	7.3
6–10	10.8	3.6	20.9
11–15	14.4	5.4	13.7
16–20	20.8	6.3	13.7
21–25	19.8	18.9	12.7
26–30	10.8	28.0	4.5
31–35	11.7	15.3	8.2
36–40	5.4	7.2	5.4
41–45	2.7	9.9	8.2
46–49	0.9	5.4	0.9
50–55	0.0	0.0	1.8
56–59	0.0	0.0	2.7

Child and parent ratings of children’s depressive symptoms did not significantly correlate with one another (Table 4). However, parent reports of their own depressive symptoms were positively correlated with parent ratings of children’s depressive symptoms; however, parent reports of their own depressive symptoms were *negatively* correlated with children’s reports of depressive symptoms. Parents’ attributional style was not significantly related to children’s reports of depressive symptoms or parents’ reports of their own depressive symptoms; however, an optimistic/neutral style was negatively associated with parents’ reports of child symptoms. Fisher’s *z*’ tests showed no differences between the correlations for males and females.

**Table 4:** Correlations between child depressive symptoms, parent depressive symptoms, and children’s coping strategies

	Child reports child symptoms	Parent reports child symptoms	Parent reports parent symptoms	Parent optimistic/neutral style	Active coping/emotion regulation	Disengagement coping
Parent report child symptoms	.02	–				
Parent report parent symptoms	− .23*	.37***	–			
Parent optimistic/neutral attributional style	− .05	− .24*	− .12	–		
Active coping/emotion regulation	.00	− .09	− .06	− .02	–	
Disengagement coping	.33**	.14	− .03	− .03	− .13	–
Seeking answers/positive thinking	− .07	− .18	− .15	− .01	.42**	− .15

*p < .05; **p < .01;  ***p < .001

Table 5 shows the results of the two regressions predicting child and parent ratings of depressive symptoms from parent depressive symptoms, parents’ optimistic/neutral attributional style, and their interaction. Both regressions were significant: child ratings, *F*(3,104) = 3.62, *p* < 0.05; parent ratings, *F*(3,104) = 7.87, *p* < 0.001. For child ratings of depressive symptoms, both the main effect of parent depressive symptoms and the interaction between parent attributional style and parent depressive symptoms were significant. When the correlations between child ratings of depressive symptoms and parent ratings of their own depressive symptoms were examined separately by parent attributional style, only the correlation for parents with a pessimistic style was significant, *r*(35) = -0.47, *p* < 0.01; the correlation for parents with an optimistic or neutral style was nonsignificant, *r*(69) = -0.04, n.s. Because the direction of this correlation was opposite of predictions, we inspected the scatterplot of this relationship for parents with a pessimistic attributional style. This showed that the association was not due to a small number of cases and that the relationship was found across all levels of parental depressive symptoms. Moreover, when we examined the partial correlation between parental depressive symptoms and children’s reports of depressive symptoms for this group statistically controlling for parental diagnosis (i.e., depression versus other), the association was still significant and negative, *r*(34) = -0.41, *p* = 0.01.

**Table 5:** Linear regressions predicting children’s and parents’ ratings of children’s depressive symptoms from parent depressive symptoms, parent optimistic/neutral attributional style, and their interaction

Predictor	Dependent variables							
	Child ratings of depressive symptoms				Parent ratings of child depressive symptoms			
	*B*	*SE*	Standardized beta	*t*	*B*	*SE*	Standardized beta	*t*
Parent depressive symptoms	− 0.30	0.09	− 0.44	− 3.24**	0.27	0.09	0.40	3.11**
Parent optimistic/neutral attributional style	1.79	1.88	− 0.09	.95	− 3.95	1.80	− .20	− 2.20*
Parent depressive symptoms × parent attributional style	0.27	0.13	0.29	2.16*	− .06	0.12	− 0.07	− 0.51

*p < .05; **p < .01

The regression predicting parent ratings of child depressive symptoms showed a different pattern. As shown in Table 5, parent depressive symptoms were *positively* associated with parent ratings of children’s depressive symptoms and the optimistic/neutral attributional style was negatively associated. The interaction in this analysis was not significant.

Table 6 presents the regressions examining the relationships between child coping strategies and child depressive symptoms controlling for parent depressive symptoms and parent attributional style. Both regressions were significant: child ratings, *F*(5,102) = 3.99, *p* < 0.01; parent ratings, *F*(5,102) = 5.54, *p* < 0.001. Inspection of the beta weights showed that, consistent with the simple correlations, that child ratings of depressive symptoms were predicted negatively by parent depressive symptoms and positively by disengagement coping; parent ratings of child depressive symptoms, in contrast, were predicted positively by parent depressive symptoms and negatively by an optimistic or neutral attributional style. When the regressions were run adding the sex by coping interaction terms for the three coping scores, no interactions were significant. The positive relationship between disengagement coping and child ratings of depressive symptoms was significant for both males, *r*(57) = 0.36, *p* < 0.01, and females, *r*(49) = 0.30, *p* < 0.05.

**Table 6:** Linear regressions predicting child and parental ratings of child depressive symptoms from parent depressive symptoms, arent optimistic/neutral attributional style, and the child coping variables

Predictor	Dependent variables							
	Child ratings of depressive symptoms				Parent ratings of child depressive symptoms			
	*B*	*SE*	Standardized beta	*t*	*B*	*SE*	Standardized beta	*t*
Parent depressive symptoms	− 0.16	0.06	− 0.23	− 2.52*	0.23	0.06	0.34	3.82***
Parent optimistic/neutral attributional style	− 1.43	1.83	− 0.07	− 0.78	− 4.02	1.78	− 0.20	− 2.26*
Active coping/emotional regulation	1.24	3.04	0.04	0.41	− 0.71	2.96	− 0.02	− 0.24
Disengagement coping	9.89	2.98	0.31	3.32***	3.25	2.91	.10	1.12
Seeking answers/ positive thinking	− 3.24	3.18	− 0.10	− 1.02	− 3.71	3.11	− 0.12	− 1.19

*p < .05;  **p < .01;  ***p < .001

## Discussion

The purpose of this study was to examine the relationships between parental depression, parental attributional style, children’s coping strategies and 5–12-year-old children’s depressive symptoms in a sample of Chinese children whose parents had been diagnosed with an anxiety or a mood disorder. Consistent with previous studies of children’s and parents’ reports of depressive symptoms in Western and Chinese societies [40, 51], the present study reported higher levels of child depressive symptoms reported by parents than did children. The first hypothesis, that children of parents with anxiety or mood disorders would show high levels of depressive symptoms, was supported. The vast majority of children in this study exceeded the clinical cut-off of 15, for both children’s and parents’ reports of depressive symptoms (72.1% for child reports and 91.0% for parent reports). These values are much higher than the value that Wong and Power [40] found for child reports in a community sample of 6-year-olds during their first year of primary school in Hong Kong (29%) and higher than the rate (13.2%) found in a survey of more than 1,300 primary school age children in Hong Kong [52]. Parent reports in the Wong and Power [40] study, although higher (45%) than the child reports, were still much lower than the 91% reported in the current sample. Consistent with studies of Western samples, there were no sex differences in the depressive symptoms of the children in this sample [5].

The percentage of children exceeding the clinical cutoff on the CES-DC in this sample (72.1%) is at the very high end of percentages reported by Downey and Coyne [19] in their summary of nine previous studies of affective disorders in children of clinically depressed mothers (21–74%). This high percentage likely results from two factors: (1) depressive symptoms were assessed with questionnaires rather than with clinical interviews (i.e., the clinical cutoffs on the CES-DC are helpful in screening for depression risk, but do not provide a clinical diagnosis) and (2) among children and adolescents, Chinese samples show higher levels of depressive symptoms than Western samples [53–55]. Therefore, although Chinese children’s depressive symptoms peak in mid-adolescence [54], our findings are consistent with multiple studies showing high levels of depressive symptoms in Chinese primary school children [53, 55].

The second hypothesis that parents’ depressive symptoms would be positively associated with child’s depressive symptoms was partially confirmed. As expected, parent reports of their own depressive symptoms were positively correlated with parent ratings of children’s depressive symptoms; unexpectedly, parent reports of their own depressive symptoms were *negatively* correlated with children’s reports of depressive symptoms. Moreover, unlike in community samples, where the correlation between parents’ and children’s ratings of child depressive symptoms is typically around *r* = 0.26 [14, 16, 40], the correlation in this study was not significant, *r* = 0.02, suggesting that the severity of the parents’ emotional problems may have made it difficult for parents in the current sample to accurately evaluate their children’s depression.

The third hypothesis was supported for parent ratings of depressive symptoms in their children—that is, parents with an optimistic or neutral attributional style rated their children as showing fewer depressive symptoms than parents with a pessimistic style. Because parents’ attributional style was not associated with children’s ratings of depressive symptoms, it is not clear whether or not an optimistic attributional style served as a protective factor against childhood depression, or whether parents with an optimistic style simply saw their children’s behavior in a more positive way. This is clearly a question for future research.

It is difficult to understand the negative relationship between parental depressive symptoms and the children’s ratings of their own depressive symptoms. This is inconsistent with the larger literature, where studies with clinic samples in Western settings show a positive relationship between parent and child depressive symptoms, *r* = 0.23 [5]. Interestingly, the test of the fourth hypothesis showed that the negative relationship in the current study was only found for parents with a depressive attributional style and that the effect remained unchanged even after controlling for parental diagnosis. One possible reason for the difference between our findings and those in the larger literature is that we were not directly comparing children of clinically depressed parents to a healthy control group; instead, we examined variation in depressive symptoms within a clinic sample. Given how high childhood depressive symptoms were in our sample, if we had a healthy comparison group, it is very likely that the mean symptoms for children of parents in the clinic group would be significantly higher than in the healthy controls, therefore, replicating previous studies of clinical populations in the literature. If our finding is not an artefact of this sample and is replicated in another Chinese clinical sample, one way to interpret this within-sample variation would be to consider how parents with high levels of depressive symptoms and a pessimistic attributional style may differ from parents with high levels of depressive symptoms and an optimistic or neutral style. Clearly, the first group is more distressed than the second and our findings showed that the more distressed parents were within this group, the fewer symptoms their children reported. It is possible that children whose parents have very high levels of distress may be less likely to report their depressive symptoms for any number of reasons (they do not want to draw attention to themselves, they see their own problems as much less serious than their parents’, their parents have told them they do not have serious problems, so on). Alternatively, these children may actually experience fewer depressive symptoms because the seriousness of their parents’ emotional problems has led them to seek out support from other individuals (e.g., the other parent, grandparents, teachers) and this support buffered the negative impact of their parent’s depression. Additionally, children of parents with high levels of distress may have normalized their family experiences (i.e., saw parental depression as normal) and this may have reduced somewhat the negative impact of parental depression. Clearly, this unexpected correlation needs to be replicated and further explored.

The final hypothesis predicted that children’s coping would be associated with their depressive symptoms. The principal components analysis of the coping questionnaire in this sample yielded slightly different components than those found in Wong and Power [28]. Rather than engagement, disengagement, and distraction components, the current analysis yielded two engagement and one disengagement components. One engagement component, active coping/emotion regulation, focused on behavioral strategies (including distraction) and the other, seeking answers/positive thinking, focused on cognitive strategies. Both components were primarily made up of secondary control strategies such as distraction, spending time with others, and positive thinking. This is not surprising given that parental depression represents a more uncontrollable stressor than the academic stressors studied by Wong and Power [28, 40] and that children tend to use secondary control strategies more often in uncontrollable situations [56].

Consistent with predictions, and as found in numerous studies in community samples [24, 25], the current results showed a significant positive relationship between children’s disengagement coping and their reports of depressive symptoms. Given the cross-sectional design, however, the direction of effects is not clear: it could be that reliance on avoidant strategies to address the stresses associated with parent depression may contribute to greater levels of depressive symptoms in this population or it could be that depression in children might lead to a greater use of avoidant strategies. It is also possible that the direction of effects is bidirectional, creating a downward spiral for children of depressed parents.

Unexpectedly, neither engagement strategy showed the predicted negative associations with child depressive symptoms. This contrasts with Wong and Power [28] who found significant negative associations between females’ use of engagement coping and depressive symptoms in coping with the transition to primary school. Children of parents with anxiety or mood disorders may need more support other than simply learning engagement coping strategies to cope with the stresses of being raised by a parent with an anxiety or mood disorder.

Finally, the hypothesis that the relationships between children’s coping strategies and depressive symptoms would only be significant for females was not confirmed. The findings for disengagement coping in this study were equally strong for males and females. This may be in part due to the high levels of child depressive symptoms found in this study, and the significant stresses of being raised by a parent with a clinically diagnosed anxiety or mood disorder.

### Limitations of This Study

This study had several limitations that could be addressed in future studies. First, the relatively small number of participants in each diagnostic group prevented us from examining possible moderating effects of parent diagnosis on the child depressive outcomes. Second, our sample included mainly mothers with anxiety or mood disorders, and therefore may not reflect the associations between father’s depressive symptoms, father’s attributional style, and children’s depressive symptoms. Moreover, we did not collect data on the mental health status of both parents, making it possible to examine the degree to which the mental health status of the other parent exacerbated or buffered the impact of having a parent with an anxiety or mood disorder on the mental health of the child. Other limitations are that we did not conduct clinical interviews with the children (instead relying on questionnaires), did not assess children’s perceptions of their parent’s depressive symptoms, and did not assess parent coping to determine if children modelled the coping strategies of their parents. Finally, the cross-sectional design prevented us from examining child depression, coping strategies, and attributional styles over time, making it impossible to draw conclusions about the direction of effects.

The study’s strengths, however, should be considered along with its limitations, including the examination of a high-risk and understudied population, the use of standardized and well-validated measures of depressive symptoms and child coping, and the use of both parent and child ratings of children’s depressive symptoms. Future studies with larger samples, multiple methods, and longitudinal designs could further examine the relationships identified here.

### Implications

The high levels of depressive symptoms found in children of Chinese parents with an anxiety or mood disorder support the need for early identification of, and intervention programs for, depressed parents and their children. The lack of a significant correlation between parents’ and children’s ratings of child depressive symptoms suggests that parents’ emotional distress may prevent them from accurately judging the level of emotional distress in their child. Interventions for depressed parents should be designed to help parents learn to identify signs of emotional distress in their children, as well as give parents strategies to help their children cope with stress. Finally, the lack of significant relationships between children’s engagement coping and their depressive symptoms suggests that ordinary coping strategies may not be sufficiently powerful to mitigate against the development of child depression in this population, suggesting that interventions should help children learn effective coping strategies for dealing with the specific stresses of living with a parent with an anxiety or mood disorder.

## Summary

The current study examined the relationships between parental depression, parental attributional style (pessimistic vs. optimistic attributional style), children’s coping strategies and children’s depressive symptoms in a sample of 5–12-year-old Chinese children whose parents had been diagnosed with an anxiety or a mood disorder. The present study confirmed that children of parents with anxiety or mood disorders would show high levels of depressive symptoms. Parents with an optimistic or neutral attributional style rated their children as showing fewer depressive symptoms than parents with a pessimistic style. This study showed a significant positive relationship between children’s disengagement coping and children’s reports of depressive symptoms. Unexpectedly, neither engagement strategy showed the predicted negative associations with child depressive symptoms. Children of parents with anxiety or mood disorders may need more support other than simply learning engagement coping strategies to cope with the stresses of being raised by a parent with an anxiety or mood disorder. The findings highlight the need for early identification of, and support and intervention programs for, parents suffering from depression and children of depressed parents as a means of protecting the psychological well-being of both parents and children.
