# Correction to: Parental Depressive Symptoms, Parent Attributional Style, and Child Coping as Predictors of Depressive Symptoms in Children of Parents with Anxiety or Mood Disorders

**DOI:** 10.1007/s10578-022-01317-8

**Published:** 2022-01-20

**Authors:** Mun Wong, Thomas G. Power

**Affiliations:** 1 Department of Early Childhood Education, The Education University of Hong Kong, 10 Lo Ping road, Tai Po, New territories, Hong Kong; 2 Department of Human Development, Washington State University, Pullman, WA USA

## Correction to: Child Psychiatry & Human Development 10.1007/s10578-021-01248-w

The article “Parental Depressive Symptoms, Parent Attributional Style, and Child Coping as Predictors of Depressive Symptoms in Children of Parents with Anxiety or Mood Disorders”, written by Mun Wong and Thomas G. Power was originally published electronically on the publisher’s internet portal (https://link.springer.com/article/10.1007/s10578-021-01248-w) on 21 September 2021 without open access. With the author(s)’ decision to opt for Open Choice, the copyright of the article changed on 12 January 2022 to © Author(s) 2022 and the article is forthwith distributed under the terms of the Creative Commons Attribution 4.0 International License (http://creativecommons.org/licenses/by/4.0/), which permits use, duplication, adaptation, distribution and reproduction in any medium or format, as long as you give appropriate credit to the original author(s) and the source, provide a link to the Creative Commons license and indicate if changes were made.

The original article has been corrected.

